# Specific microRNAs for Modulation of Autophagy in Spinal Cord Injury

**DOI:** 10.3390/brainsci12020247

**Published:** 2022-02-11

**Authors:** Rhett Visintin, Swapan K. Ray

**Affiliations:** 1 Department of Chemistry and Biochemistry, University of South Carolina, Columbia, SC 29208, USA; rhettv@email.sc.edu; 2 Department of Pathology, Microbiology, and Immunology, University of South Carolina School of Medicine, Columbia, SC 29209, USA

**Keywords:** spinal cord injury (SCI), autophagy, neurodegeneration, miRNAs, miRNAs for modulation of autophagy, neuroprotection, functional recovery

## Abstract

The treatment of spinal cord injury (SCI) is currently a major challenge, with a severe lack of effective therapies for yielding meaningful improvements in function. Therefore, there is a great opportunity for the development of novel treatment strategies for SCI. The modulation of autophagy, a process by which a cell degrades and recycles unnecessary or harmful components (protein aggregates, organelles, etc.) to maintain cellular homeostasis and respond to a changing microenvironment, is thought to have potential for treating many neurodegenerative conditions, including SCI. The discovery of microRNAs (miRNAs), which are short ribonucleotide transcripts for targeting of specific messenger RNAs (mRNAs) for silencing, shows prevention of the translation of mRNAs to the corresponding proteins affecting various cellular processes, including autophagy. The number of known miRNAs and their targets continues to grow rapidly. This review article aims to explore the relationship between autophagy and SCI, specifically with the intent of identifying specific miRNAs that can be useful to modulate autophagy for neuroprotection and the improvement of functional recovery in SCI.

## 1. Introduction

**Figure 1:**
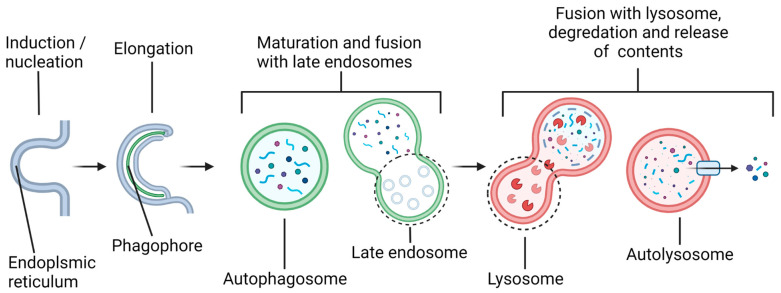
Overview of the autophagy pathway. Induction of autophagy begins with the formation of a cup-shaped domain of the endoplasmic reticulum called an omegasome. The nascent autophagosome, called a phagophore or induction membrane, is formed from the omegasome, and it grows until it has sealed around its cargo and produced a mature autophagosome. The autophagosome subsequently fuses with a late endosome, also known as multivesicular bodies (MVBs), ultimately merging with a lysosome or lysosomes. Acid hydrolases in the lysosome degrade the inner autophagosomal membrane and its contents into the cellular building blocks, which, afterwards, are released into the cytosol for recycling in the cell.

Alteration of the status of autophagy via microRNAs (miRNAs) may profoundly influence the course of pathogenesis and locomotor recovery in spinal cord injury (SCI). Our increasing understanding of the biochemical mechanisms of autophagy and miRNAs and their interactions in preclinical models of SCI will open new therapeutic avenues for successful treatment of the patients who become the victims of this devastating neurological condition. Autophagy is a general term that can refer to several distinct cellular processes, including macroautophagy, chaperone-mediated autophagy (CMA), and microautophagy. This review article focuses specifically on macroautophagy, which will hereafter be referred to as “autophagy”. Autophagy is utilized by a cell to break down and recycle its numerous substrates, either in “bulk autophagy”, targeting essentially random volumes of cytoplasmic contents, or in “selective autophagy”, targeting specific substrates, including protein aggregates, organelles such as mitochondria, parts of the nucleus, invading bacteria, proteasomes, peroxisomes, lysosomes, and others [1]. Over 40 proteins that make up the core autophagy machinery, known as autophagy-related (ATG) proteins, are highly conserved among eukaryotes [2,3] and have been most extensively studied in yeast, though analogs in several model organisms, as well as humans, have been identified [1]. Autophagy involves several steps (Figure 1), beginning with the formation of a double-membrane vesicle (called an autophagosome) from a nucleating membrane (called the phagophore or induction membrane) at sites on the endoplasmic reticulum known as omegasomes [4].

As the phagophore expands, it envelops cargo destined to be degraded after maturation of the autophagosome and fusion with a lysosome(s). The vesicle resulting from this fusion is called an autolysosome, and it is here that lysosomal acid hydrolases degrade the autophagosomal contents into their basic units (amino acids, nucleotides, etc.), which are subsequently released back into the cytosol for recycling. The autophagy pathway is active at the basal level to maintain cellular homeostasis but is also activated by several stress signals, such as an increase in the AMP/ATP ratio due to starvation (via activation of the 5′ AMP-activated protein kinase or AMPK) or excessive reactive oxygen species (ROS) produced by the defective mitochondria, among others [5,6]. The total degradative throughput due to autophagy in the cell is called autophagic flux. The genetic or pharmacological modulation of autophagic flux has been implicated in numerous pathophysiological conditions, including cancers, neurodegenerative diseases, and SCI [7,8,9].

Acute SCI is a debilitating condition, which exerts significant physical, psychological, and economic tolls on the SCI patients, their families, and the healthcare system. The incidence of traumatic SCI is about 54 per 1,000,000 people (17,900 new cases per year) in the United States, at a total cost of around $15.7 billion dollars per year [10]. Complications and prognosis depend on the location and severity of the injury, with cervical and thoracic SCI associated with poorer and poor outcomes, respectively [11]. Some potential complications of SCI include partial or full paralysis below the injury level; cardiovascular issues; autonomic dysreflexia; joint degradation and/or ossification; reduced respiratory function; chronic pain; and sexual, urinary, and gastrointestinal dysfunctions, among others [12]. Due to the low innate regenerative ability of the spinal cord, recovery in SCI patients is usually incomplete, and the current therapies are limited in their efficacy [11]. Due to this grim situation, therapies targeting neuroprotection and neuroregeneration after SCI are an active area of research [13,14]. Due to its prominent role in cellular homeostasis, cellular stress responses, and programmed cell death, the modulation of autophagy presents a promising avenue for the treatment of SCI.

## 2. Different Phases of Traumatic SCI and Its Complex Pathophysiology

**Figure 2:**
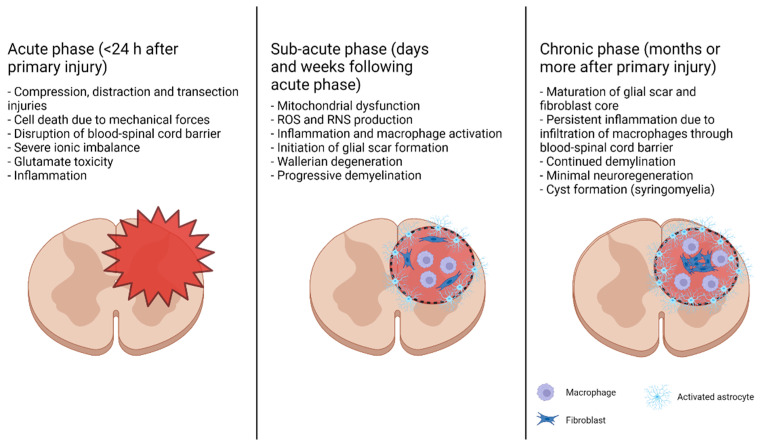
Secondary phases of SCI. After the primary traumatic injury (compression, distraction, transection, etc. of the spinal cord), the acute secondary phase immediately follows. Mechanical force ruptures cells and disturbs the blood–spinal cord barrier, allowing entrance of the blood-born immune cells into the lesion site. Glutamate excitotoxicity and disruption of the ionic balance led to mass necrosis. After about 24 h, the subacute phase begins, marked by the initiation of an astrocyte-based glial scar around the lesion. The damaged mitochondria release damaging ROS, causing the axons near the lesion site to begin to demyelinate and die back. In the chronic stage, the glial scar matures, and a fibroblastic core is formed. The blood–spinal cord barrier remains disrupted, leading to chronic inflammation. Axon demyelination and degeneration continues. The presence of the scar chemically and physically hinders neuroregeneration.

The progression of traumatic SCI is divided into two major phases: primary and secondary injuries. Primary injury is usually sudden and occurs due to mechanical forces (such as injury from a car accident or fall), resulting in fractures in the vertebrae and compression, distraction, and/or transection of the spinal cord [15,16]. A primary injury to the spinal cord triggers a secondary injury, which is characterized by a signaling cascade creating a microenvironment that results in further damage to the spinal cord and inhibits neuroregeneration. Secondary injuries can be further divided into acute (minutes to hours after primary injury), subacute (days to weeks after primary injury), and chronic phases (weeks to months after primary injury), though these phases are not clearly delineated and may overlap (Figure 2). Acute secondary injury is characterized by events including ischemia–reperfusion injury, the death of cells due to mechanical insult, necrosis resulting from glutamate excitotoxicity and ionic imbalance, and inflammation due to the activation of resident microglia and astrocytes and exacerbated by the introduction of blood-born immune cells due to permeation of the blood–spinal cord barrier [15,17,18]. In the subacute phase, mitochondrial dysfunction due to an increased intracellular Ca^2+^ load and immune cell activation result in the production of ROS and reactive nitrogen species (RNS), damaging proteins, nucleic acids, and lipid membranes; these events subsequently cause the induction of apoptosis in and around the primary injury site or the lesion site. The activation of macrophages and death of oligodendrocytes lead to axonal demyelination and Wallerian degeneration [19]. Inflammation triggers the beginning of glial scar formation around the lesion perimeter by the activated reactive astrocytes. The chronic phase of SCI involves glial scar maturation through the formation of a pericyte/fibroblast-derived fibrotic core surrounded by an astrocytic outer layer, the formation of cysts (syringomyelia), and continued apoptosis and demyelination.

Neuroregeneration during the subacute and chronic phases is inhibited by several factors. Debris from degraded oligodendrocytes and myelin contains three myelin-associated inhibitors, such as myelin-associated glycoprotein (MAG), oligodendrocyte-myelin glycoprotein (OMgp), and Nogo-A, and the activators of the small GTPase RhoA and its downstream effector Rho kinase (ROCK) [20]. Activation of the RhoA-ROCK signaling pathway is associated with cell death and the loss of neural processes and synapses, causing perturbation of the information flow in the spinal cord. Activation of the RhoA-ROCK signaling pathway subsequently destabilizes cytoskeletal elements in neurite growth cones, leading to their collapse and the formation of dystrophic end bulbs [20]. Thus, inhibition of the RhoA-ROCK signaling pathway appears as a promising approach for treating SCI. Reactive astrocytes and oligodendrocyte precursor cells in the glial scar act as a physical and chemical barrier to neural regeneration, as they express neuroregeneration-inhibitory factors into the extracellular matrix, including chondroitin sulfate proteoglycans, which also activate the RhoA-ROCK signaling pathway [15,21]. Incomplete healing of the blood–spinal cord barrier (BSCB) leads to persistent macrophage infiltration into the lesion site, causing chronic inflammation [19,22]. Taken together, the complex interactions of the numerous factors that may promote or inhibit neuroregeneration depending on their localization and evolution over time indicate that a multifaceted approach to the treatment of SCI will be necessary to maximize the functional recovery.

## 3. Biogenesis and Roles of miRNAs in the Context of SCI

Various studies have indicated roles of miRNAs in neurodegeneration, as well as in neuroprotection, following SCI in preclinical models. Our goal is the exploration of specific miRNAs that increase the autophagy flux for neuroprotection in SCI. All miRNAs are short, noncoding RNA sequences (about 22 nucleotides long when mature), which have a significant regulatory effect on the post-transcriptional gene expression in many organisms by targeting and silencing mRNA transcripts. Since the initial identification of miRNAs in *Caenorhabditis elegans* nearly 30 years ago, our understanding of the scope of their influence has increased greatly [23,24,25,26]. Some miRNAs found in humans are evolutionarily conserved [27]. At least 60% of human genes are regulated by miRNAs, and there are virtually no human biological pathways that are not in some way affected by miRNAs [28]. Specific miRNAs play significant roles in the modulation of autophagy flux for the prevention of pathogenesis and promotion of neuroregeneration in SCI.

**Figure 3:**
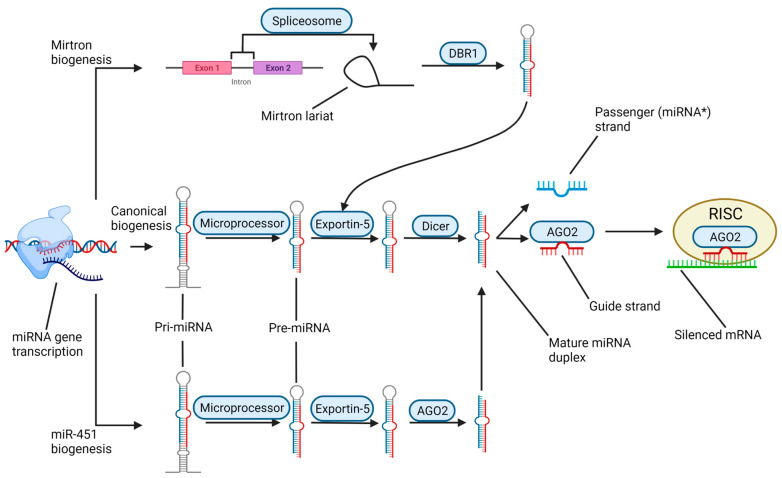
Biogenesis of matured miRNA. The first step for all pathways is the transcription of a miRNA gene by RNA Pol II. In the canonical pathway, the newly formed pri-miRNA hairpin is trimmed by the microprocessor complex in the nucleus to form a pre-miRNA, which is then transported out of the nucleus by Exportin-5. In the cytosol, Dicer cleaved the stem–loop of the pre-miRNA to form the mature miRNA duplex. The guide strand is separated from the passenger strand (miRNA*) and loaded onto Argonaute 2 (AGO2). AGO2 joins the RNA-induced silencing complex (RISC), where the target mRNA is selected by base-pairing with the loaded miRNA. Depending on the completeness of the match, the target mRNA may be either cleaved by AGO2 or be blocked from ribosomal translation. In the noncanonical pathway, Mirtrons are formed from introns during the splicing process. After forming a Mirtron loop, the nascent miRNA is debranched by the DBR1 and processed in the nucleus to form a pre-miRNA, which is then transported out of the nucleus by Exportin-5 and subsequently follows the canonical pathway. The biogenesis of miR-451 is unique in that it is independent of Dicer; after being translated, processed by a microprocessor, and transported out of the nucleus, it binds directly to AGO2, which cleaves it into a mature miRNA duplex.

We will briefly describe the biogenesis of miRNAs and how they cause the silencing of mRNAs. Biogenesis begins with the transcription of a miRNA gene by RNA polymerase II (Pol II), though the transcription and subsequent processing can vary widely (Figure 3). In the canonical pathway, miRNA genes have their own promoters and are transcribed as pri-miRNAs; these genes may be located within or between protein-coding genes, and they may overlap introns, exons, and/or lncRNA genes [29]. Several clustered miRNA genes may be translated as a single polycistronic pri-miRNA. The relatively long pri-miRNA transcript subsequently folds into one or more hairpin structures before being processed in the nucleus by the microprocessor, a protein complex including the RNase III Drosha and two dimerized DiGeorge Critical Region 8 (DGCR8) proteins [30]. A microprocessor cleaves the pri-miRNA, leaving a ~60–70-nucleotide-long hairpin known as pre-miRNA. The pre-miRNA is transported out of the nucleus by Exportin-5 and into the cytoplasm, where it is further processed by Dicer, another RNase III-type protein, which cleaves the molecule near the loop to produce mature double-stranded miRNA.

There are multiple forms of noncanonical miRNA biogenesis, which differ from the canonical pathway by skipping either the Drosha-regulated or Dicer-regulated steps [31]. Mirtrons are miRNA precursors formed by the spliceosome during mRNA intron removal, and they require debranching by the RNA debranching enzyme 1 (DBR1) but they are Drosha-independent. There is a single known Dicer-independent miRNA, miR-451, which is Drosha-dependent but subsequently processed directly by Argonaute 2 (AGO2) instead of Dicer.

All miRNA biogenesis pathways converge at the point where the mature miRNA duplex is loaded onto AGO2, which is a part of the RNA-Induced Silencing Complex (RISC). The guide strand is then separated from the passenger strand (miRNA*), which is then degraded; the selected strand determines the specificity for the downstream silencing target [32]. The 5′ nucleotide of each strand (with a preference for adenosine) and the thermodynamic stability of the duplex ends determine which strand becomes the guide strand [33]. The identification of mRNA targets of RISC occurs primarily by complementary base pairing between the 5′ seed region of the guide miRNA (nucleotides 2–8) and the 3′ untranslated region (3′ UTR) of the mRNA [34]. The mechanism of silencing of mRNA depends on the extent of base pairing between the seed region and the target mRNA sequence; exact matches result in cleavage of the mRNA by AGO2, while partial matches result in translation repression [27].

The above description makes it clear how the structure of the matured miRNAs use the mechanisms to target and repress mRNAs of specific genes for affecting intracellular signaling pathways. A recent study showed that increasing the expression of a specific miRNA could target and repress the mRNA of the mechanistic target of rapamycin (mTOR), an inhibitor of autophagy, to sustain neuroprotective autophagy and promote motor function recovery in SCI [35]. In this study, investigators increased the level of miR-421-3p that could bind to the 3’ UTR of mTOR to significantly reduce mTOR activity and increase autophagy and decrease neuronal apoptosis in SCI mice. Further, treatment with an miR-421-3p inhibitor showed a decrease in neuroprotective autophagy in SCI mice.

## 4. Components of the Autophagy Pathway from Nucleation to Fusion

We are somewhat tersely describing different components of the complex autophagy pathway for an appreciation of its mechanism for a neuroprotective role in SCI. The autophagy pathway is divided into four phases: nucleation (or induction) of the phagophore, elongation of the phagophore, maturation of the autophagosome, and fusion of autophagosome with a lysosome to form an autolysosome. Regulation of autophagy flux in SCI depends on the spatiotemporally precise activity of numerous ATG proteins. Initiation of the nucleation phase depends on the recruitment and activation of two protein complexes: the Unc-51-like autophagy activating kinase (ULK) complex and the class III phosphatidylinositol-3-kinase complex (PIK3C3) [4,36].

The ULK complex is comprised of Unc-51-like serine/threonine kinases 1 and 2 (ULK1 and ULK2 [37,38]), the focal adhesion kinase (FAK) family kinase-interacting protein of 200 kDa (FIP200), ATG13, and ATG101. The ULK complex acts as a gatekeeper for the autophagy pathway; it receives information about the status of the cell from its upstream regulators—most significantly, the mTOR complex 1 (mTORC1) and AMPK—and modulates the autophagy flux accordingly [39].

AMPK is an important sensor of metabolic stress in the cell, and it acts to inhibit and activate numerous anabolic and catabolic pathways, respectively. In addition to the “canonical” activation of AMPK due to phosphorylation by the tumor suppressor liver kinase B1 (LKB1) and increase in the AMP levels, AMPK may also be activated by other indicators of cellular stress, such as glucose starvation and Ca^2+^ release from endoplasmic reticulum (ER), leading to AMPK phosphorylation [40]. Subsequently, AMPK phosphorylates proteins associated with the cell growth–cell survival axis (including the ULK complex), resulting in an increase in catabolic processes to provide the cell with the resources needed to adapt to changing conditions [41]. Thus, AMPK acts as an activator of the ULK complex and positively regulates autophagy.

The serine/threonine protein kinase mTOR forms two protein complexes: mTORC1 and mTORC2. mTORC1 is a complex of the mTOR catalytic subunit, Raptor (regulatory-associated protein of mammalian target of rapamycin; necessary for proper localization of the complex to the lysosomal surface and recognition of some substrates [42,43]), PRAS40 (Proline-Rich Akt Substrate of 40 kDa), DEPTOR (DEP domain containing the mTOR-interacting protein), the Tti1/Tel2 complex, and mLST8 (mammalian Lethal with SEC13 protein 8) [44]. mTORC1 is activated in response to increases in the cellular nutrient and energy levels [40,45]. Activated mTORC1 facilitates cell growth by upregulating anabolic processes such as protein and lipid biosynthesis and by downregulating catabolic processes like autophagy via phosphorylation of the ULK complex [46].

AMPK also regulates mTORC1. The inhibition of mTORC1 by AMPK is achieved by direct phosphorylation of the Raptor subunit of mTORC1, as well as by phosphorylation of the tuberous sclerosis complexes (TSC) 1 and 2 (TSC1/2), which are GTPase-activating proteins (GAP) affecting the Rheb–GTP complex, an activator of mTORC1 [44,47]. Additionally, mTORC1 inhibits the nuclear localization of TFEB (Transcription Factor EB) and TFE3 (Transcription Factor binding to IGHM Enhancer 3), translation factors promoting lysosome biogenesis and autophagy, while AMPK activity is necessary for the activation of those same factors [48,49]. Furthermore, the interactions between AMPK/mTORC1 and the ULK complex are not unidirectional. ULK1 can phosphorylate and inhibit both mTORC1 and AMPK, resulting in seemingly contradictory positive and negative feedback loops, respectively [50,51,52].

**Figure 4:**
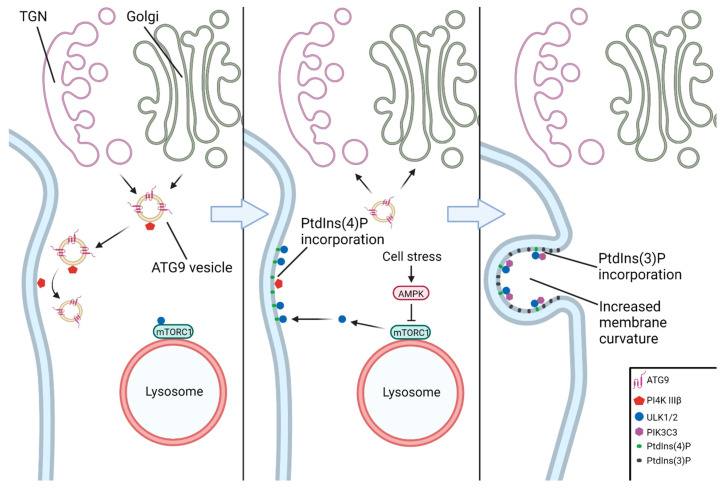
A model of formation of the omegasome. ATG9 vesicles carry PI4K IIIβ from the Golgi and trans-Golgi network to the omegasome formation site. PI4K IIIβ enriches the ER membrane in PtdIns(4)P, which binds ULK1/2 after its AMPK-mediated release from mTORC1 at the lysosomal surface. ULK1/2 subsequently recruits PIKC3C, which enriches the ER membrane in PtdIns(3)P, resulting in increased membrane curvature. As more PtdIns(3)P is incorporated, the highly curved membrane forms the omegasome, where the phagophore may be nucleated.

The earliest event in the nucleation of the phagophore is generally considered to be the recruitment of the ULK complex to a nascent nucleation site on the ER (Figure 4). The ATG9A–vesicle-mediated incorporation of PtdIns(4)P (phosphatidylinositol-4-phosphate) into the ER membrane by PI4K IIIβ (phosphatidylinositol-4-kinase III beta) facilitates binding of the ULK complex to the nucleation site [53,54]. The ULK complex then activates PIKC3C, which subsequently enriches the ER membrane with PtdIns(3)P (phosphatidylinositol-3-phosphate), leading to the formation of a cup-shaped ER subdomain known as an omegasome [55,56]. The formation of omegasomes and subsequently autophagosomes has been shown to be associated with MCS (membrane contact sites), the most well-described being the ER–phagophore and ER–mitochondria contact sites (as in mitophagy) [57,58,59].

**Figure 5:**
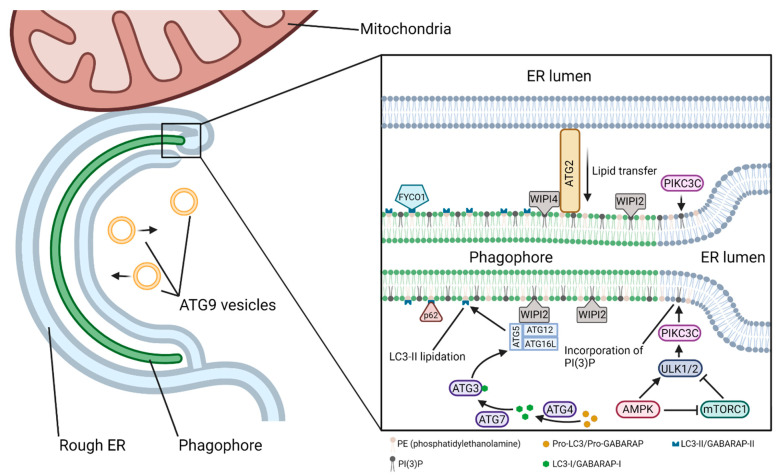
Elongation of the phagophore. WIPI proteins are recruited to the phagophore membrane by PtdIns(3)P, where they act as an adaptor for a variety of proteins that shape and modify the phagophore. The membrane sources that feed the growing phagophore can be varied. Direct extrusion of the ER or other membranes may contribute to phagophore growth. Additionally, ATG2 may transfer lipids from one membrane to another, such as from the ER or ATG9 vesicles (not shown). ATG9 vesicles may also donate lipids through a kiss-and-run mechanism, though no ATG9 proteins are transferred to the phagophore. Pro-LC3 in the cytosol is converted by ATG4 into LC3-I, which is transported to ATG3 by ATG7. ATG3 brings LC3-I to the ATG12–ATG5–ATG16L1 complex, which processes it into LC3-II and conjugates it to PE (phosphatidylethanolamine) in the phagophore membrane. LC3-II then acts as an adaptor for various effector proteins.

The beginning of the elongation phase is marked by the recruitment of members of the WIPI (WD-repeat domain phosphoinositide-interacting protein) family of proteins to the nascent phagophore (Figure 5). All four of the WIPI proteins (WIPI1–4) involved in the autophagy pathway are effectors of the PtdIns(3)P produced by PIKC3C at the omegasome [60]. WIPI1 and WIPI2 are the first to associate with the phagophore, and while not strictly necessary for autophagy, WIPI1 has been shown to associate with and enhance the action of WIPI2 [61]. WIPI2 subsequently recruits the ATG12–ATG5–ATG16L1 complex, which is necessary for the lipidation of LC3 (microtubule-associated protein Light Chain 3)/GABARAP (GABA type A Receptor-Associated Protein) family proteins to the inner and outer autophagosomal membranes [62,63,64]. LC3 lipidation is accomplished through a ubiquitin-like conjugation system. Pro-LC3/pro-GABARAP in the cytosol is cleaved by ATG4 to form LC3-I/GABARAP-I, which is then activated by and transferred to ATG7 (E1-like) and subsequently transferred to ATG3 (E2-like). Finally, the ATG12–ATG5–ATG16L1 (E3-like) complex promotes the conjugation of LC3-I/GABARAP-I to PE (phosphatidylethanolamine) in the autophagosomal membrane to form LC3-II/GABARAP-II [65]. The LC3-II/GABARAP-II family proteins are crucial for autophagosomal growth and closure, cargo targeting through adaptor proteins such as sequestosome 1 (SQSTM1)/p62 (in selective autophagy), and maturation [66]. LC3-II is a reliable marker of autophagosome formation.

Growing phagophores are continuous or closely associated with membranes of the ER, Golgi complex (GC), recycling and late endosomes (LEs), lysosomes, mitochondria, and plasma membrane, as well as lipid droplets [58,67,68]. The ER, which is in contact with both the inner and outer surfaces of the phagophore [69,70], along with the mitochondria, can act as the primary suppliers of lipids to the phagophore, whether by membrane extrusion or the direct movement of lipids across lipid–transfer proteins such as the ATG2–WIPI4–ATG9 complex [71,72]. Though the ER acts as an inexhaustible lipid pool, phagophore-localized *de novo* production of lipids by ER-resident lipid synthesis proteins is a significant contributor to phagophore expansion [73,74]. ERGIC (ER-Golgi intermediate compartment)-derived COPII (cytoplasmic coat protein complex II) vesicles and recycling endosome-derived vesicles positive for ATG9 and ATG16L1 contribute to, or even serve as the platform for, phagophore initiation and/or growth [4,53,75,76,77,78].

Phagophore at an appropriate size must be sealed to form an autophagosome. The sealing is facilitated by the ESCRT (endosomal-sorting complexes required for transport) machinery (consisting of protein complexes ESCRT-0 through ESCRT-III). Of these, only ESCRT-III is strictly necessary to produce the sealed autophagosomes, though the autophagic flux is hindered in the absence of ESCRT-0, ESCRT-I, and ESCRT-II [79,80,81,82,83]. Recruitment of the ESCRT machinery to unsealed phagophores may follow the canonical pathway [83] or an alternate pathway, as in yeast [84]. The closure is completed when ESCRT machinery is disassembled by the VPS4 (vacuolar protein sorting 4) complex.

Autophagosome maturation necessitates the removal of ATG proteins from the outer membrane, primarily by cleavage/delipidation of ATG8 members by the ATG4 family of proteases but also potentially by the activity of PtdIns(3)P phosphatases [85,86,87]. An autophagosome undergoes fusion with a lysosome to form an autolysosome, where lysosomal hydrolases degrade the inner autophagosomal membrane and its contents into basic units (amino acids, nucleotides, etc.) to be reused by the cell. Before fusion with a lysosome, mature autophagosomes may fuse with early and/or late endosomes (LEs) to form amphisomes, and there is evidence that this may, in fact, be required for fusion with lysosomes [87,88]. The fusion of autophagosomes with lysosomes/LEs involves several SNARE (Soluble N-ethylmaleimide-sensitive factor Attachment protein REceptor) complexes, motor proteins, tether complexes, Rab GTPases, and their GEFs (guanine nucleotide exchange factors). To begin with, the autophagosomes must be brought into contact with lysosomes, which are localized near the nucleus under starvation [89]. The positioning of autophagosomes and lysosomes (and the resulting autophagic flux) is influenced by interactions with the motor proteins dynein and kinesin, which are mediated by PtdIns(3)P, ATG8 members, and RAB7 (RAs-related in Brain protein 7, a member of small GTPases) in autophagosomes and ARL8 (ARf-Like protein 8), BORC ((BLOC-One-Related Complex), RILP (Rab Interacting Lysosomal Protein), RAB7, ORP1L (oxysterol-binding protein or OSBP-Related Protein 1L), and others in (auto)lysosomes [87,89,90,91].

Subsequently, tether complexes link the two membranes for fusion by binding PtdIns(3)P, ATG8 members, components of the SNARE complex, and/or RAB7 or its effectors. The primary contributor is the HOPS (HOmotypic fusion and vacuole Protein Sorting) complex, though EPG5 (Ectopic P-Granules autophagy protein 5 homolog), ATG14L, and BRUCE (Baculovirus IAP Repeat-containing Ubiquitin-Conjugating Enzyme) may play roles in fusion [92,93]. The tethering factors promote the recruitment of autophagy-associated SNARE complexes, of which there are two in humans: the YKT6 (vesicular SNARE homolog)–SNAP29 (SyNaptosomal-Associated Protein 29)–STX7 (SynTaXin 7) complex and the STX17-SNAP29-VAMP7/8 (Vesicle-Associated Membrane Proteins 7 and 8) complex [94,95]. Additionally, there is evidence that STX16 plays a role in fusion [96]. Fusion releases the inner autophagosomal membrane and its contents into the interior of the lysosome, where they are degraded by acid hydrolases and released into the cytosol for recycling and cell survival.

Recent studies show the important roles of some specific miRNAs in the suppression of selective mRNAs for the promotion of autophagy and inhibition of apoptosis, providing us unique therapeutic opportunities for neuroprotection and functional recovery in many preclinical models of SCI [97,98]. The therapeutic use of specific miRNAs for the promotion of protective autophagy is likely to inhibit inflammation and neuropathic pain, prevent apoptosis in neurons and glial cells, and contribute to axonal regeneration mostly in the remote regions of the central nervous system (CNS) injuries [97,98,99].

## 5. Specific miRNAs in Modulation of Autophagy in Preclinical Models of SCI

Autophagy at the basal levels is generally understood as promoting cell survival, but under certain conditions, changes in the autophagy flux or blockage of the autophagy pathway can promote cell death. Autophagy-dependent cell death in a strict sense appears to be a highly specific process primarily occurring during early development [100]. What occurs in many pathophysiological conditions like SCI is more accurately called autophagy-associated or autophagy-mediated cell death, wherein changes in the autophagy flux accompany and interact with the activation of other cell death pathways such as apoptosis. The crosstalk between autophagy and apoptosis is complex and an area of active research. There are several enlightening review papers already published on the subject [101,102,103]; however, some of the different ways autophagy and cell death are related will be briefly covered here for some context.

A buildup of autophagosomal bodies in cells has been shown to precede apoptosis in neurons and other cells [101,102,104]. Such a buildup may be the result of an increased production of autophagosomes and/or inhibition of autophagosome clearance. There is evidence that autophagosomal membranes act as scaffolds for the assembly of pro-apoptotic and pro-necrotic protein complexes [101]. An increased number of autophagosomes may therefore induce cell death by promoting the formation of these complexes. Defective autophagy can lead to the accumulation of protein aggregates as well as of damaged mitochondria, which subsequently can cause mitochondrial release of cytochrome c into the cytosol to initiate apoptosis [105]. Several ATG proteins are known to become pro-apoptotic factors when cleaved by calpain (ATG5) or caspases (ATG4 and Beclin 1) [101,102]. Selective autophagy may target either pro-death or pro-survival factors to influence the fate of a cell [106,107]. The exact nature of the interactions among the autophagy, apoptosis, and necrosis that determine cell death or survival are still not well understood.

**Figure 6:**
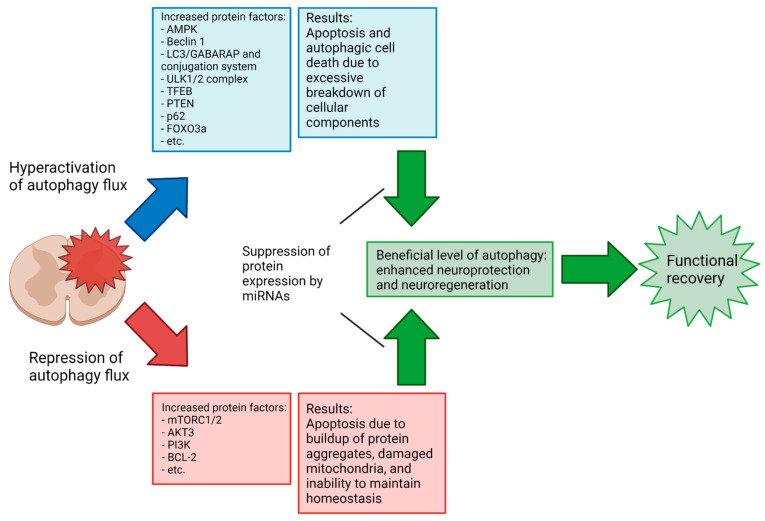
Potential role of miRNAs in promoting functional recovery in SCI. Autophagy may be hyperactivated or repressed to non-optimal levels at a given phase of the SCI secondary injury, leading to poorer recovery or even itself causing deleterious effects. An understanding of the effects that autophagy has on different SCI types and at different phases of secondary injury will be vital to developing effective therapies.

Apoptotic activity following SCI varies significantly depending on the type and severity of the injury, as well as over the phases of SCI recovery [9,108]. However, a consistent difficulty in assessing the overall efficacy of autophagy activation/inhibition at a given phase in SCI recovery results from a lack of consensus in the literature on the results of autophagy activation or inhibition in a given injury context; a recent meta-analysis demonstrated that SCI recovery is improved by the modulation of autophagy but shows no significant difference in the Basso, Beattie, and Bresnahan (BBB) locomotor scores between upregulation and downregulation in mouse injury models [109]. In the previously referenced meta-analysis, the 33 included studies generally achieved a modulation of autophagy in SCI by chemical means (e.g., rapamycin and metformin) rather than via miRNAs. In comparison, the number of studies examining the modulation of autophagy in SCI by miRNAs specifically is small, and to the authors’ knowledge, no comparable meta-analysis exists. Notably, there is a lack of studies in the literature examining the miRNA-mediated upregulation of autophagy in SCI (miR-15a is the sole miRNA with sufficient previous research to be included in our review that caused an increase in autophagy flux). As previously noted, the complexity and sensitivity of autophagy regulation pathways and the double-edged nature of autophagy’s relation to cell death suggest that this inconsistency in results may be due to small differences in the experimental procedure, and further research is needed to elucidate the points at which the neuroprotective or neurodegenerative properties of autophagy dominate (Figure 6). The effort toward full elucidation of the autophagy’s role in SCI across various injury types and phases might benefit from more standardized research methodologies regarding injury type and how long after the primary injury the intervention occurs. Due to the highly dynamic characteristics of SCI, comparisons of the results that arise from experimental conditions that are not highly similar are of limited value. Increased collaboration between research groups in the field to develop standardized methodologies to allow more meaningful comparisons of results could help resolve some of the seemingly conflicting conclusions so far obtained.

With that in mind, the following sections will explore the relationship between autophagy and SCI, with a particular focus on specific miRNAs that may modulate these interactions for neuroprotection in preclinical models of the SCI (Table 1). Most of the investigations in this field are currently focused on the fine tuning of interactions between specific miRNAs and the molecular components of autophagy, as we described above, to regulate the autophagy flux for functional neuroprotection in preclinical models of SCI with a hope to apply this approach to the SCI patients as soon as possible.

### 5.1. Autophagy in Neurons

Neuronal autophagy is critical for homeostasis in the CNS, as neurons are post-mitotic and therefore limited in their ability to deal with cellular waste. Additionally, their unique morphology creates the requirement for specialized autophagy processes not found in other cell types. Autophagosome formation is constitutively activated not just in the soma but occurs as far as the axon terminal, allowing for a rapid autophagy response along the axon [110]. Degradation of the autolysosomal contents takes place primarily in the soma, necessitating that the kinesin/dynein-mediated anterograde movement of lysosomes and retrograde movements of autophagosomes and autolysosomes along the cytoskeleton be tightly regulated due to the long distances involved [111,112]. The movement of autophagosomes through the axon appears to be strictly unidirectional; somatic autophagosomes are barred from entering the axon [113]. Finally, the LC3-II levels in neurons are relatively low in basal conditions; whether this indicates an innately lower rate of autophagosome formation or a more rapid flux rate in neurons is currently unclear [114].

**Table 1:** Specific miRNAs in modulating autophagy with prospect of influencing SCI recovery.

microRNA	Molecular Target(s)	Effect on Autophagy Flux	Prospect of SCI Recovery	References
miR-93-5p	PTEN	Decrease	Beneficial	[115,116,117]
	ATG7			
	TLR4			
miR-384-5p	Beclin 1	Decrease	Beneficial	[118,119]
	GRP78			
miR-378	ATG12	Tissue dependent	Beneficial	[120,121,122,123]
	GRB2			
miR-27a	FOXO3a	Decrease	Beneficial	[124,125,126,127]
	DRAM2			
	PINK1			
miR-223	RPH1/KDM4A	Decrease	Beneficial	[128,129]
miR-124	PI3K	Decrease	Beneficial	[130,131,132]
	AMPK			
	Bcl-2			
	p62			
miR-212-3p	PTEN	Decrease	Beneficial	[133,134,135,136]
miR-15a	Akt3	Increase	Beneficial (in neuropathic pain model)	[137,138]
	Rictor			
miR-384-5p	Beclin 1	Decrease	Beneficial	[139]
miR-223	FOXO3a ATG16L	Decrease	Beneficial	[140,141,142,143,144]
miR-30	Beclin 1	Tissue dependent	Context dependent	[145,146,147,148,149]
miR-30d	Beclin 1	Increase or decrease	Beneficial	[150,151,152]

In rat retinal ganglion neurons, miR-93-5p was found to reduce autophagy-associated cell death after N-methyl-D-aspartate (NMDA)-induced excitotoxicity by downregulating the expression of phosphatase and tensin homolog (PTEN), which negatively regulates autophagy through the Akt/mTOR pathway [115]. ATG7 and Toll-like receptor 4 (TLR4, an inducer of neuronal autophagy and neuroinflammation [116]) have also been demonstrated to be targets of miR-93-5p, as the overexpression of miR-93-5p results in significantly reduced levels of those proteins, with an accompanying reduction in autophagy and inflammatory factors and increase in cell survival in rat myocardial tissue [117].

miR-384-5p is one of several miRNAs that target the expression of Beclin 1 (an inducer of autophagy), which contributes to functionality of the PI3K complex [118]. The suppression of Beclin 1 therefore halts autophagy at the point of PtdIns(3)P enrichment and prevents phagophore elongation. Specifically, the application of miR-384-5p was shown to significantly improve the BBB locomotor scores in rats with spinal cord compression injuries after 7 days and 21 days as compared to untreated rats [119]. The same study also reported that inhibition of ER stress via decreasing levels of glucose-regulating protein 78 (GRP78), an ER stress response-mediating protein that is also targeted by miR-384-5p, may have contributed to the lower level of autophagy seen in the study [119].

It has been demonstrated that miR-378 regulates the expression of several ATG proteins and appears to have a promising prospect for the modulation of autophagy in neurons. A report indicated that miR-378 negatively regulated ATG12 expression in rat neurons and reduced apoptosis when administered immediately after contusion SCI, with a corresponding improvement of the BBB locomotor scores 7 days post-injury compared to the sham group, though the study did not identify whether the improvement was necessarily due to autophagy impairment [120]. Studies on the long noncoding RNA (lncRNA)-based inhibition of miR-378 revealed that it acts to inhibit autophagy by targeting growth factor receptor-bound protein 2 (GRB2) and ATG12 [121,122]. Alternatively, one study has found that miR-378 activates autophagy in skeletal muscle, indicating that further research into how miR-378 functions in different cell types and what conditions are necessary [123].

Multiple studies have implicated miR-27a in modulating neurodegenerative processes through the modulation of autophagy. It has been reported that the overexpression of miR-27a downregulates autophagy in neurons and inhibits neurodegeneration by targeting FOXO3a (Forkhead bOX O3a, a transcription factor that promotes autophagy [124]) in mouse neurons after a traumatic brain injury [125]. Another study found that miR-27a also targets damage-regulated autophagy modulator 2 (DRAM2) and that the rno-circRNA_010705 (circLrp1b)-mediated repression of miR-27a can increase autophagy-associated neurodegeneration [126]. Additionally, miR-27a has been shown to downregulate mitophagy in HeLa cells by targeting PTEN-induced kinase 1 (PINK1), which is a mitochondrial serine/threonine kinase, to suggest that it may protect cells from excessive mitochondria loss in hyper-autophagic conditions [127].

RPH1 (Repressor of PHR1)/KDM4A (lysine- or K-specific DeMethylase 4A) is a DNA-binding protein that acts as a histone demethylase and negatively regulates the transcription of several ATG genes. It has been reported that miR-223 targets RPH1/KDM4A, subsequently reducing the autophagy levels in lipopolysaccharide-treated neuronal PC-12 cells and attenuating cell death [128]. Another study reported that, in yeast, RPH1/KDM4A acted as an autophagy inhibitor under nutrient-rich conditions and did not show any effect on translation in nitrogen-starved conditions [129]. Clearly, more research is necessary to clarify the functions of RPH1/KDM4A, specifically in mammalian cells, to rectify this difference.

The expression of miR-124, a key miRNA in neural development, has been linked to regulation of the autophagy pathway via its regulation of the Akt/AMPK/mTOR axis [130]. However, as in many other cases involving autophagy, there is conflicting evidence as to whether its overexpression or under-expression bestows neuroprotective and/or neuroregenerative effects in the spinal cord specifically. A study found that antagomiR-124 had a neuroprotective effect when introduced 5 days before spinal cord ischemia–reperfusion injury in rats, with a reduction in apoptosis and increase in the levels of mitochondrial LC3-II and Beclin 1, causing an increase in neuronal mitophagy [131]. Conversely, another report showed that antagomiR-124 administered 24 h before cerebral ischemia–reperfusion injury produces a neuroprotective effect by promoting the PI3K/Akt/mTOR pathway, with rats exhibiting an increase in the PI3K/Akt/mTOR levels and improvement in neurological function, in part, due to downregulation of autophagy by the PI3K/Akt/mTOR pathway [132]. To reconcile these conclusions, it should be noted that these studies involved neural tissue in different locations and quantified different proteins (PI3K/Akt/mTOR function upstream of LC3-II and Beclin 1 in the autophagy pathway) in different cell fractions (the whole cell vs. mitochondria). The conflicting results from these studies underline the need for more thorough research into the function of this specific miR-124 as it relates to pathophysiology of SCI before any potential therapy utilizing it is developed.

In a recent study, miR-212-3p has been shown to have a positive effect on recovery from SCI in rats [133]. PTEN is a possible target of miR-212-3p, and it is demonstrated that the silencing of PTEN may be the reason behind the improvement in the recovery in SCI rats treated with miR-212-3p. The molecular mechanism involved in this recovery process seems so far convincing. The silencing of PTEN leads to the activation of Akt and mTOR, suggesting that the beneficial effects of miR-212-3p may be due to the inhibition of autophagy. Indeed, other studies have linked miR-212-3p to autophagy regulation in cardiomyocytes, osteosarcoma cells, and prostate cancer cells [134,135,136]. However, it has yet to be conclusively shown that the neuroprotective effects of miR-212-3p in SCI are due to autophagy inhibition.

### 5.2. Autophagy in Glial Cells

A study shows that miR-15a positively regulates autophagy in rat microglial cells by reducing the Akt3 levels and increasing the expression of ATG proteins [137]. In addition, another study showed that miR-15a and miR-16 promote autophagy in HeLa cells by targeting the Rictor subunit of mTORC2, an upstream activator of Akt3 [138].

In addition to the previously mentioned role of miR-384-5p beneficially downregulating autophagy in neurons, there is evidence that it has anti-inflammatory effects in the brain through the inhibition of autophagy in macrophages [139]. The pro-inflammatory effects of autophagy in this case may be due to autophagy leading to an increase in the macrophage cell viability, which is negated by suppression of autophagy promoter Beclin 1 by overexpression of miR-384-5p that binds to 3’ UTR of Beclin 1 mRNA [139]. If so, this would highlight one of the ways that the modulation of autophagy in each direction might have significantly different effects (e.g., cell survival vs. cell death) in different cell types, including those that might be closely associated in the context of a particular injury.

As previously mentioned for its role in neural autophagy, miR-223 has also been implicated in reducing neuroinflammation in microglia by targeting ATG16L [140,141]. Interestingly, it has been found that FOXO3a is yet another autophagy-related target of miR-223, with a negative correlation between the miR-223 levels and FOXO3a expression reported in several cell types [142,143,144].

Various studies have clearly indicated that Beclin 1 in the CNS tissues is a potential target of several members of the miR-30 family [145,146,147,148,149]. Of these, the effects of miR-30d are the best characterized in relation to SCI. AntagomiRs of miR-30d promoted autophagy and reduced apoptosis in post-ischemia astrocytes [150] and neonatal rat neurons [151], while another study demonstrated that miR-30d decreased neuroinflammation by reducing autophagy in macrophages and subsequently promoting M2 macrophage polarization over M1 [152]. Additionally, the sponging of miR-30b and miR-30d by lncRNAs SNHG12 (small nucleolar RNA host gene 12) and C2dat2 (CAMK2D-associated transcript 2), respectively, was shown to increase autophagy and apoptosis after cerebral ischemia–reperfusion injury [146,153].

## 6. Conclusions

Autophagy plays a significant role in influencing the pathogenesis and outcomes in SCI, but the current treatments do not address this critical factor, leaving plentiful opportunities to discover novel therapies for a devastating neuropathological condition that affects the lives of many people, who are mostly young individuals. Interest in the involvement and use of specific miRNAs to treat numerous conditions continues to grow, and recent research has identified many miRNAs that may regulate autophagy in the pathophysiological processes in the CNS [154,155]. Both miRNAs and autophagy are associated with ER stress, which triggers the unfolded protein response (UPR) to restore normal function of the ER [156]. Future studies therefore need to identify the molecular mechanisms of how specific miRNAs interrupt the UPR for the induction of autophagy under ER stress in SCI. Clearly, many uncertainties remain. The role of autophagy as either a neuroprotective or neurodegenerative factor in the various phases of SCI has not been fully elucidated, with the current studies exhibiting a lack of consensus at any given point. Additionally, while there are numerous studies on the effects of autophagy and its modulation by miRNAs in the CNS, there are relatively few focusing specifically on SCI, which has a unique biomolecular environment. The current investigations indicate that miRNA therapies are an attractive and promising prospect for the treatment of SCI, but a greater understanding of the process of autophagy and its effects in the spinal cord, as well as the further identification of potentially beneficial miRNAs and elucidation of the overall effects of specific miRNAs in the regulation of autophagy in the context of pathophysiology of SCI, will be necessary before any effective therapies can be developed in the future.

## Data Availability

It is not applicable.
